# Shock assisted ionization injection in laser-plasma accelerators

**DOI:** 10.1038/srep16310

**Published:** 2015-11-09

**Authors:** C. Thaury, E. Guillaume, A. Lifschitz, K. Ta Phuoc, M. Hansson, G. Grittani, J. Gautier, J.-P. Goddet, A. Tafzi, O. Lundh, V. Malka

**Affiliations:** 1 LOA, ENSTA ParisTech, CNRS, École Polytechnique, Université Paris-Saclay, 828 bd des Maréchaux, 91762 Palaiseau France; 2 Department of Physics, Lund University, P. O. Box 118, S-22100 Lund, Sweden; 3 Institute of Physics ASCR, v.v.i. (FZU), ELI Beamlines project, Na Slovance 2, 18221 Prague, Czech Republic; 4 Czech Technical University in Prague, FNSPE, Brehova 7, 11519 Prague, Czech Republic

## Abstract

Ionization injection is a simple and efficient method to trap an electron beam in a laser plasma accelerator. Yet, because of a long injection length, this injection technique leads generally to the production of large energy spread electron beams. Here, we propose to use a shock front transition to localize the injection. Experimental results show that the energy spread can be reduced down to 10 MeV and that the beam energy can be tuned by varying the position of the shock. This simple technique leads to very stable and reliable injection even for modest laser energy. It should therefore become a unique tool for the development of laser-plasma accelerators.

Laser plasma wakefield accelerators can produce femtosecond electron beams^1^ with energies up to a few GeV^2^,^3^,^4^, in only a few centimeters. A critical issue with plasma accelerators is to trap electrons into the wakefield in a reliable and controllable way. Ionization injection is one of the most effective methods for achieving this trapping^5^,^6^,^7^,^8^,^9^. This technique is based on the use of a high-Z gas or a gas mixture (low and high Z gases). While outer shell electrons of the high-Z gas are ionized by the rising edge of the laser pulse, inner shell electrons are released only when the laser reaches its peak intensity. At that time, a wakefield cavity is already formed. Electrons from inner shells can therefore be released in the middle of the cavity. They are then accelerated as they slip toward the back of the cavity and possibly injected if their velocity exceeds the wakefield velocity. This injection technique allows to accelerate electron beams with high charge^10^ and low emittance^11^ but large energy spread^6^,^7^,^8^.

This large energy spread comes from the fact that the laser field remains above the threshold for inner shell ionization over a significant length (possibly a few millimeters), leading to continuous electron injection. Several techniques have been proposed to control the trapping length and hence the energy spread in ionization injection. They include the use of moderate power laser pulses^12^, multiple beams setups^5^,^13^,^14^,^15^, and plasma density tailoring^16^ notably in staged laser-plasma accelerators^17^,^18^,^19^. Yet, energy spreads obtained in experiments are at best about 10 MeV^17^, which remains relatively large compared to best controlled injection techniques^20^,^21^.

Here we propose and demonstrate a controlled ionization injection technique which is simpler to set up than existing ones and leads to mean energy spreads of about 10 MeV, with room for improvement. It is based on the creation of a shock front in a supersonic gas jet. Electrons ionized when the laser crosses the shock front spend more time in the accelerating field because the cavity expands, as illustrated in Fig. 1. They can thus be injected below the threshold for regular ionization injection, leading to localized trapping and low energy spreads. This technique is quite similar to density-transition injection^22^, except for the use of a gas mixture instead of a pure light gas. We show in the following that this simple change stabilizes the injection and improves the beam quality significantly.

## Results

**Figure 1:**
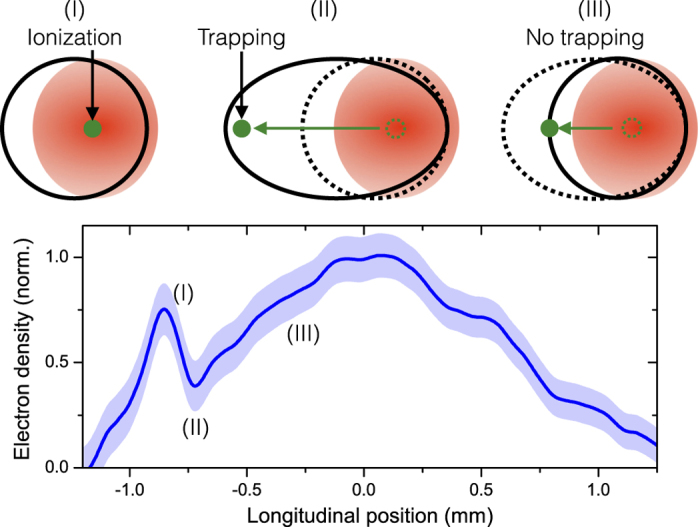
Principle of shock assisted ionization injection and typical density profile. Electrons ionized close to the center of the plasma cavity when the laser crosses the density transition (between I and II) spend a long time in the accelerating field due to the expansion of the cavity in the shock front. Therefore, they are much more likely to be trapped than electrons ionized in the up-ramp density gradient (III) which have to catch up a shrinking (accelerating) cavity. For low enough laser intensity and plasma density the injection can thus be restricted to the shock front. The shaded area indicates the standard error on the measurement. Note that the measured shock front is not fully resolved; it may actually be significantly sharper and denser (the resolution is about 40 *μ*m).

The experiment was performed at LOA using the *salle jaune* laser facility. The laser duration and focal spot were 28 fs and 12.2 × 15.7 *μ*m^2^ full width at half maximum (FWHM) respectively. The peak laser intensity in vacuum was *I* = (5 ± 2) × 10^18^ W cm^−2^. The shock front was formed by inserting a 500 *μ*m thick silicon wafer into the supersonic gas flow from a 1.5 mm nozzle, 3 mm from the nozzle exit. Its position along the laser propagation axis was varied in order to tune the electron beam energy. The density profile was characterized at every shot by plasma interferometry and Abel inversion. Note that the shock front is almost perpendicular to the optical axis within the plasma (which radial extension is about 160 *μ*m FWHM), Abel inversion can therefore be performed using the optical axis as a symmetry axis. A typical density profile is shown in Fig. 1. Unless otherwise stated, the gas is a mixture composed of 99% of helium and 1% of nitrogen. Helium is fully ionized in the rising edge of the laser, while nitrogen is ionized to *N*^5+^ ; electrons from upper levels can be ionized when the laser reaches its peak intensity, leading to ionization injection. Electron spectra were measured with a spectrometer consisting of a permanent magnet (1.1 T with a length of 100 mm) combined with a phosphor screen imaged on a 16 bit CCD camera. The spectral resolution varies between 2.7% and 3.8% for electron energy between 75 and 200 MeV. All measurement precisions were obtained from the standard deviation.

**Figure 2:**
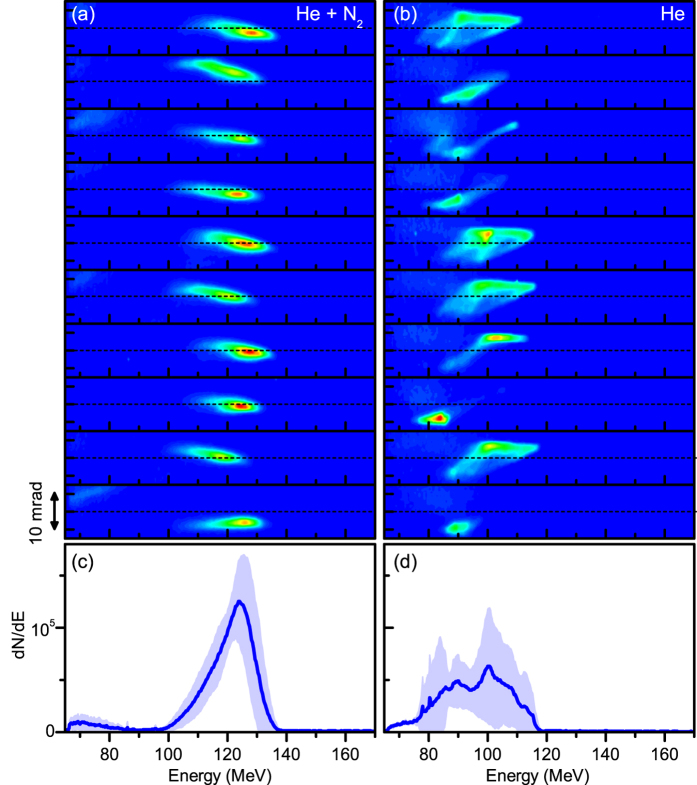
Angularly resolved electron spectra of ten consecutive shots, with a shock front and a gas mixture (**a**) or pure helium (**b**). The electron density at the center of the density profile is 4 ± 0.1 × 10^18^ cm^−3^ in (**a**) and 3 ± 0.1 × 10^18^ cm^−3^ in (**b**). The shock front is located 1.03 ± 0.02 mm from the center of the density profile in (**a**) and 1.34 ± 0.02 mm in (**b**). The small differences in density and shock front position explain the difference in the electron beam energy between (**a**,**b**). The spectra are corrected from the spectrometer dispersion. The color bar goes from 0 (blue) to 83000 electrons per MeV per mrad (red). (**c**,**d**) Corresponding mean electron spectra, with standard deviation in colored area.

Ten consecutive electron spectra obtained with a shock and a gas mixture are shown in the left side of Fig. 2. Electrons are injected at every shot (over hundreds of consecutive shots). The beam charge is about 1 pC. The mean energy spread is 14 ± 2 MeV (11% at 123 MeV), while the energy spread of the best shots is about 10 MeV. This is much smaller than typical energy spread obtained with regular ionization injection, as exemplified in Fig. 5d. The accelerated beam is elliptic^9^ with a divergence of 5 ± 0.6 mrad in the laser polarization direction and 2.6 ± 0.7 mrad in the perpendicular direction. The beam stability is compared with that of shock front injection in pure helium in Fig. 2. The charge and the energy spread are similar in both cases. In contrast, the stability is much better in the gas mixture; the standard deviation of the electron beam energy and charge are 2.5% and 12% respectively, while in pure helium they are 7% and 24%. The use of gas mixture improves also significantly the rms pointing stability; it is 1.5 mrad in the gas mixture versus 3.2 mrad in pure helium (in other series the rms pointing stability is as low as 0.7 mrad and it is never larger than 1.8 mrad with the gas mixture). Beam fluctuations in pure helium are likely due to a deficient laser wavefront^23^, to inhomogeneities in the laser spot or to imperfect shock fronts. In the gas mixture, ionization injection restricts the injection close to the optical axis^7^,^9^; therefore it produces an electron beam which is less sensitive to radial inhomogeneities, similar to longitudinal injection^24^. Accordingly, shock assisted ionization injection was also observed to depend much less on shock and laser conditions than regular shock injection; for many conditions, there was no injection with pure helium while electrons were injected at every shot with the gas mixture.

**Figure 3:**
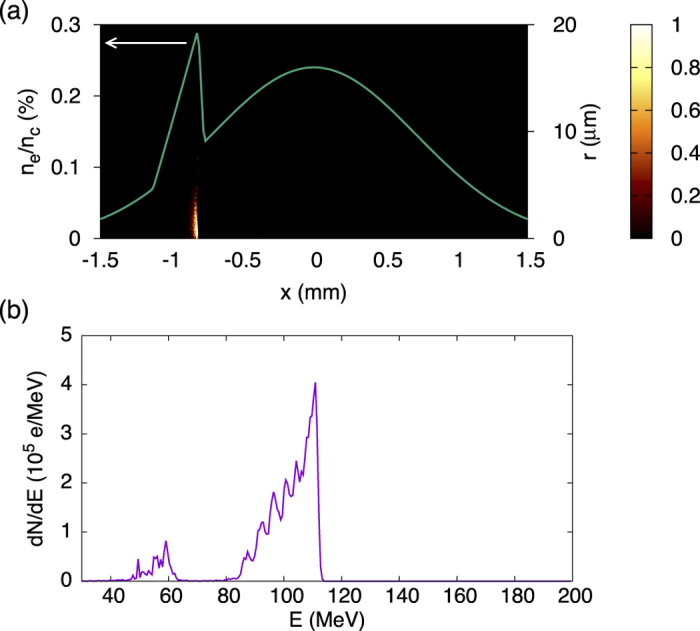
Simulation of ionization injection in a shock front. (**a**) Density profile (left axis) and initial radius of trapped electrons (right axis). The color scale indicates the density of trapped electrons in arbitrary unit. (**b**) Corresponding electron spectrum.

Particle-In-Cell simulations were used to get some insight into the injection mechanism. They were performed with the 3D, fully electromagnetic code CALDER-CIRC which uses cylindrical coordinates and a Fourier decomposition in the poloidal direction^25^. Two Fourier modes were used with 70 macro-particles by cell, and mesh sizes 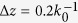 and 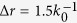 in the longitudinal and radial directions respectively, with *k*_0_ the laser wavenumber. The laser intensity was *I* = 3.3 × 10^18^ W cm^−2^ and the focal spot radius was 16 *μ*m FWHM. Ionization was described using a modified ADK model^26^. The density profile used in the simulation is shown in Fig. 3a. Because of moderate plasma density and laser intensity, the laser weakly self-focuses; the peak intensity does not exceed *I* = 1.5 × 10^19^ W cm^−2^ and a wakefield is efficiently excited over ≈1.5 mm only (from *x* ≈ −0.7 mm). Yet, an electron beam with a 1 pC charge is trapped and accelerated up to ≈110 MeV, with an energy distribution similar to the experimental one (Fig. 3b). Figure 3a shows the ionization position of accelerated electrons. Most of them originate from a short region just before the density transition. They gain some energy from the wakefield before the shock front. Then, they shift back towards the center of the plasma cavity in the density transition, because of the cavity expansion; they are thus further accelerated and gain enough energy to be trapped. Note that the peak around 55 MeV in Fig. 3b is due to regular ionization injection occurring well after the density transition; similar peaks were also observed in the experiment.

**Figure 4:**
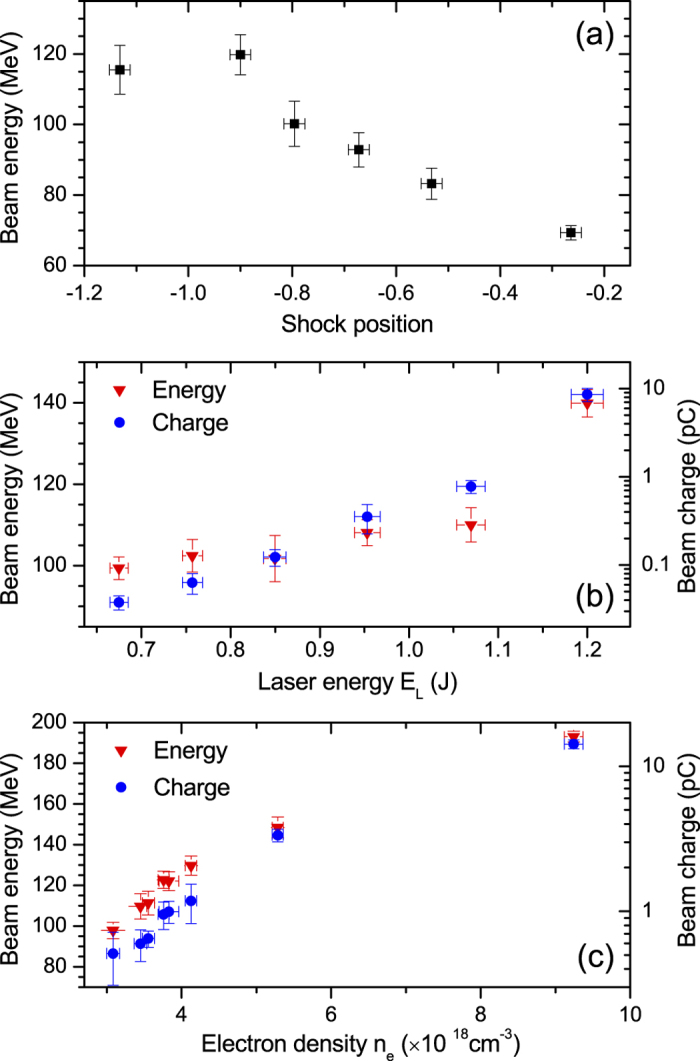
Influence of the shock position, the laser energy and the electron density on the beam properties. (**a**) Peak energy as a function of the shock position, for *n*_*e*_ = 5.3 ± 0.4 × 10^18^ cm^−3^. (**b**) Electron beam energy and charge as a function of the laser energy, for *n*_*e*_ = 5.6 ± 0.3 × 10^18^ cm^−3^. (**c**) Beam charge and energy as a function of the electron density. In (**b**,**c**) the shock position is *z*_*s*_ = −1.02 ± 0.02 mm. Error bars indicate the standard deviation.

In addition to improving the stability, shock assisted ionization injection preserves most of the advantages of shock front injection, notably its simplicity (single stage, single laser pulse) and the possibility to change the electron energy by varying the shock position. For instance, in Fig. 4(a) the energy increases from 70 MeV to 120 MeV when the shock position is moved from −0.26 mm to −0.9 mm from the center of the density profile. The charge and energy spread are almost constant, except for *z* = −0.26 where they are reduced by a factor of 10 and 4 respectively (probably because of a smoother shock front). From Fig. 4(a), the mean longitudinal field and the effective acceleration length are estimated to be about 67.5 ± 8 GeV/m and 1.65 ± 0.32 mm respectively. This length is much smaller than the dephasing length, indicating that acceleration is limited by laser diffraction, as observed in the simulation. The longitudinal field is also relatively weak, pointing out that the accelerator is operated in the quasi-linear regime, consistently with the moderate laser intensity.

Figure 4(b) shows that electrons can be injected even with modest laser energy, down to ≈670 mJ. While the electron beam energy increases slightly with the laser energy *E*_*L*_ for 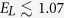 J, the rise is most significant for larger energies, likely because of better laser self-focusing and hence longer acceleration length. The beam charge (integrated over two FWHM) increases more steadily with *E*_*L*_, from 38 ± 7 fC up to 8.6 ± 1.5 pC. This increase goes along a growth of the energy spread from 9 ± 1 MeV up to 25 ± 4 MeV. These findings are consistent with injection ionization close to the injection threshold (the injected volume in the phase space increases with the laser intensity).

The charge and the energy of the beam also depend on the electron density *n*_*e*_, as shown in Fig. 4c. From *n*_*e*_ = 3.1 × 10^18^ cm^−3^ to *n*_*e*_ = 9.2 × 10^18^ cm^−3^, the charge and energy increase from 0.5 ± 0.2 to 14.2 ± 1 pC and from 98 ± 4 to 193 ± 3 MeV respectively. The charge growth arises likely from the increase of the nitrogen density and of the laser intensity (resulting from self-focusing). The beam energy increases because of rising accelerating field originating from the increase of the density and enhanced laser self-focusing. It confirms that the acceleration is not limited by dephasing (in the opposite case, the energy would decrease with increasing density).

**Figure 5:**
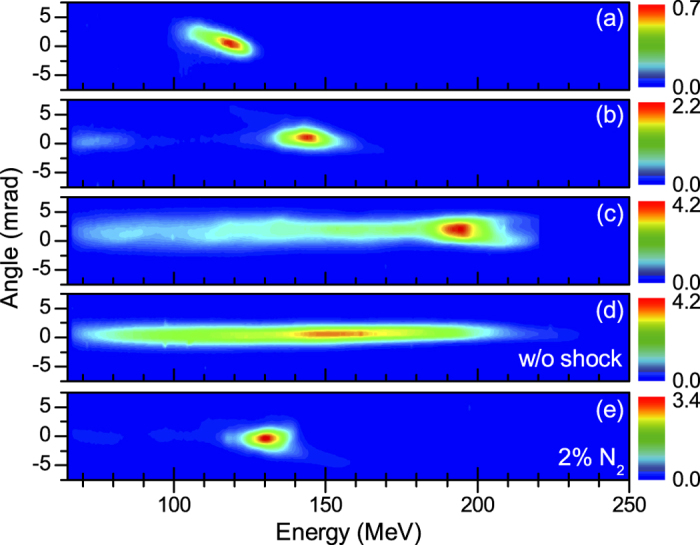
Angularly resolved electron spectra for different conditions. (**a**) Electron density *n*_*e*_ = 3.7 × 10^18^ cm^−3^. (**b**) *n*_*e*_ = 5.3 × 10^18^ cm^−3^. (**c**) *n*_*e*_ = 9.2 × 10^18^ cm^−3^. (**d**) *n*_*e*_ = 9.2 × 10^18^ cm^−3^, without shock. (**e**) *n*_*e*_ = 5.3 × 10^18^ cm^−3^, mixture 2% nitrogen, 98% helium.

As the plasma density increases some electrons can be released after the density transition, leading to injection over a long length and hence to broad energy distributions. This effect is illustrated in Fig. 5. Whereas all the charge is concentrated in a single peak at low density (Fig. 5a), a second peak with a large energy spread is also accelerated when the density is risen above ~5.3 × 10^18^ cm^−3^ (Fig. 5b,c). Note that at the threshold density (Fig. 5b), a second beam is not trapped at each shot. In contrast, for *n*_*e*_ ~ 9.2 × 10^18^ cm^−3^ the spectra presents always a low energy tail (Fig. 5c). The energy spread of the tail is very similar to that of electron beams obtained without shock at the same density (Fig. 5d), confirming that this tail is due to regular ionization injection. The total charge is also similar with or without the shock, about 30 pC, probably because of beam loading. Thus, the drawback of rising the plasma density to increase the beam charge is that it can also increase the charge in the low energy tail. A way to increase the beam charge while avoiding this detrimental effect is to rise the fraction of nitrogen in the gas mixture. This is exemplified in Fig. 5b–e which shows that doubling the proportion of nitrogen leads to the injection of more electrons in the electron beam without changing significantly the energy distribution nor the divergence.

## Discussion

In conclusion, we demonstrated a controlled injection technique which gathers most of the advantages of ionization and shock front injections. It is easy to setup and works for a large range of parameters without requiring a complex alignement. Compared to ionization injection, electron trapping is confined to a small region, leading to the injection of electron beams with rather low energy spreads (down to 10 MeV). The electron beams were also observed to be much more stable than those obtained by shock front injection. In particular the pointing stability was as low as 0.7 mrad. Further optimization of the shock may lead to higher charge and to smaller energy spread, down to 5 MeV as obtained with density-transition injection^20^. Moreover, a laser-plasma lens could be used to reduce the divergence below 2 mrad^27^,^28^.

## Additional Information

**How to cite this article**: Thaury, C. *et al.* Shock assisted ionization injection in laser-plasma accelerator. *Sci. Rep.* **5**, 16310; doi: 10.1038/srep16310 (2015).
